# PB2-588 V promotes the mammalian adaptation of H10N8, H7N9 and H9N2 avian influenza viruses

**DOI:** 10.1038/srep19474

**Published:** 2016-01-19

**Authors:** Chencheng Xiao, Wenjun Ma, Na Sun, Lihong Huang, Yaling Li, Zhaoyong Zeng, Yijun Wen, Zaoyue Zhang, Huanan Li, Qian Li, Yuandi Yu, Yi Zheng, Shukai Liu, Pingsheng Hu, Xu Zhang, Zhangyong Ning, Wenbao Qi, Ming Liao

**Affiliations:** 1 National and Local Joint Engineering Laboratory for Medicament of Zoonosis Prevention and Control, College of Veterinary Medicine, South China Agricultural University, Guangzhou, Guangdong Province 510642, People’s Republic of China; 2 Department of Diagnostic Medicine/Pathobiology, Kansas State University, Manhattan, KS 66502, USA

## Abstract

Human infections with avian influenza H7N9 or H10N8 viruses have been reported in China, raising concerns that they might cause human epidemics and pandemics. However, how these viruses adapt to mammalian hosts is unclear. Here we show that besides the commonly recognized viral polymerase subunit PB2 residue 627 K, other residues including 87E, 292 V, 340 K, 588 V, 648 V, and 676 M in PB2 also play critical roles in mammalian adaptation of the H10N8 virus. The avian-origin H10N8, H7N9, and H9N2 viruses harboring PB2-588 V exhibited higher polymerase activity, more efficient replication in mammalian and avian cells, and higher virulence in mice when compared to viruses with PB2-588 A. Analyses of available PB2 sequences showed that the proportion of avian H9N2 or human H7N9 influenza isolates bearing PB2-588 V has increased significantly since 2013. Taken together, our results suggest that the substitution PB2-A588V may be a new strategy for an avian influenza virus to adapt mammalian hosts.

Influenza A virus (IAV) is an important zoonotic pathogen that infects mammals and birds and induces seasonal influenza epidemics and pandemics in humans. IAV is a negative-sense, single-stranded, segmented RNA virus that belongs to the family *Orthomyxoviridae*. Antigenic drift (mutation) and antigenic shift (reassortment) are responsible for the rapid evolution of IAV, resulting in influenza outbreaks cannot easily be controlled despite annual vaccination efforts in human and animal populations^1^,^2^. The major natural host species of IAV are aquatic birds, in which 16 hemagglutinin (HA) and 9 neuraminidase (NA) subtype viruses are circulating, representing a large IAV gene pool^3^. The 1957, 1968, and 2009 IAV pandemics are directly related to avian influenza viruses (AIV) that provide genes resulting in generation of pandemic influenza viruses^4^. Normally, avian influenza viruses do not infect humans directly due to differences in affinity for the host receptors. Since the first human infection with a highly pathogenic H5N1 influenza virus was reported in Hong Kong in 1997^5^,^6^, this virus subtype has caused more than 800 human infections with 50% case fatality rate (WHO). AIV infection of humans usually causes conjunctivitis, mild respiratory disorders, acute respiratory distress syndrome (ARDS), and even death^7^,^8^. The cross of species barriers to humans by AIV poses a significant threat for public health since most people do not have existing immunity to emerging influenza viruses^5^,^9^.

The first human infection with an H7N9 virus occurred in March 2013, in eastern China^10^. By February 23, 2015, 571 confirmed H7N9 human infections had been reported to the WHO, with a 37% case fatality rate. On December 17, 2013, the first human infection with a novel H10N8 avian influenza virus was reported in Nanchang, Jiangxi Province^11^. Subsequently, another 2 cases were identified and 2 of these 3 H10N8-infected patients died due to severe pneumonia and complications. There are several common features between these H7N9 and H10N8 viruses. Both viruses are novel reassortants in which their HA and NA genes most likely originated from a virus in ducks or wild birds while their six internal genes (PB2, PB1, PA, NP, M, NS) are derived from endemic H9N2 influenza viruses circulating in poultry^12^,^13^,^14^. The human infections are closely associated with the local live poultry market (LPM), and the closure of LPM seems to be an effective control measure^15^,^16^. Both viruses still retain preference for avian receptors, although some human H7N9 isolates have acquired the ability to bind to human receptors^17^,^18^,^19^,^20^.

Adaption mutations in viral proteins are important strategies for AIV to cross species barriers and adapt to mammalian hosts^21^. The best example is the influenza virus polymerase subunit PB2 in which several mutations have been identified as contributing to the virulence and adaptation in mammalian hosts. One well-characterized mutation in PB2 is the glutamate change to lysine at position 627 (E627K)^22^ that results in efficient virus replication in mammalian cells and enhanced virulence in mammals^23^,^24^,^25^. This mutation is found in several subtypes of AIVs including H5N1, H7N7, and H9N2 viruses. Another well-known substitution D701N in PB2 is primarily found in a duck H5N1 virus that was highly pathogenic to mice^26^,^27^. This mutation promotes H7N7 virus replication in mammalian cells by strengthening the interaction between PB2 and importin-α1, thereby increasing the transport of PB2 into the nucleus^28^. In addition, the mutation S714R coupled with 701 N in PB2 increases the polymerase activity of the avian H5N1 influenza virus in human cells and enhances virulence in mice^29^. Several other residues, including 147 T, 339 T, 526 R, and 588 T were also found to play essential roles in avian H5N1 influenza virus virulence in mice^30^,^31^. Recent studies indicate that the residues 627 K and 701 N in PB2 are important for virulence in mammalian hosts of the H7N9 virus^32^,^33^,^34^,^35^. By contrast, the adaptation mechanisms of AIV, including the recently emerged H7N9 and H10N8 viruses are less well understood.

We previously characterized two H10N8 isolates, a human-origin strain A/Jiangxi-Donghu/346-1/2013 (JX346) isolated from a 73 year-old woman who died from severe pneumonia^11^, and an avian-origin strain, A/chicken/Jiangxi/102/2013 (JX102), that had significant homology to JX346 and was isolated from swab specimens of healthy chickens in an LPM that the patient had visited^12^. Here, we evaluated the pathogenicity of these two viruses in a mouse model and tried to understand why the H10N8 virus is able to cross species barriers to infect people. We show that the human isolate is highly pathogenic whereas the avian isolate is nonpathogenic in mice. Several residues, 87E, 292 V, 340 K, 588 V, 648 V, 676 M, together with 627 K in PB2, are responsible for their differential impact on viral virulence. The single substitution A588V in PB2 enhanced polymerase activity, virus replication, and virulence of avian influenza H10N8, H7N9, and H9N2 virus isolates.

## Results

### Characterization of avian and human H10N8 and H7N9 isolates *in vitro* and *in vivo*

**Figure 1:**
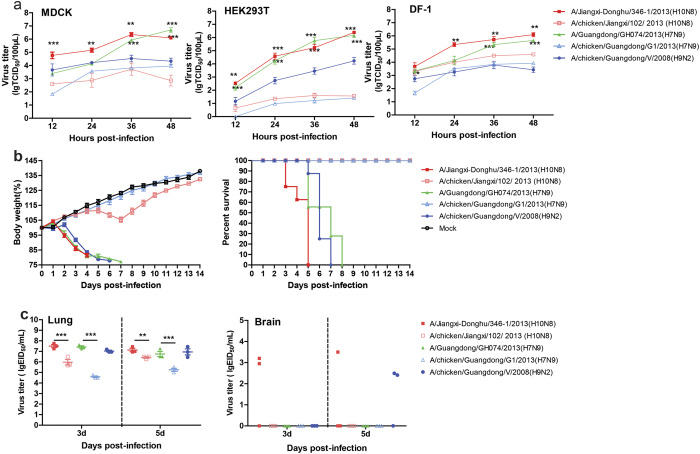
Growth kinetics and pathogenicity of H10N8 and H7N9 viruses. (**a**) Growth kinetics of H10N8, H7N9, and H9N2 viruses in cultured cells. Confluent MDCK, HEK293T, and avian DF-1 cells were infected with the indicated viruses at a multiplicity of infection (MOI) of 0.01. Virus titers were determined by using the 50% tissue culture infectious dose (TCID_50_) assay. The virus titers are means ± standard deviations (SD) (n = 3). Statistical difference between the human viruses compared with their respective avian-origin viruses were labeled (*P < 0.05, **P < 0.01, ***P < 0.001). (**b,c**) Pathogenicity and virus replication of H10N8, H7N9, and control H9N2 viruses in mice. Group of mice were intranasally infected with 10^6^ EID_50_ of each virus or mock-infected with PBS. Body weight and case fatality rate are shown in (**b**) and virus replication in mouse lungs and brains on 3 and 5 dpi is shown in (**c**). The percentage weight loss or gain from each group and each time point are expressed as means ± SD of eight infected mice. Virus titers in tissues were determined using EID_50_ assays and each datapoint represents the virus titer from an individual animal (**P < 0.01, ***P < 0.001).

To investigate the growth kinetics and virulence of the human and avian H10N8 and H7N9 viruses, a genetically-related H9N2 A/chicken/Guangdong/V/2008 (V) that is lethal in mice^36^ was used as a control. Both H7N9 A/Guangdong/GH074/2013 (GH074) isolated from a patient, and A/chicken/Guangdong/G1/2013 (G1) isolated from healthy chickens at an LPM were studied. The human-origin H10N8 JX346 and H7N9 GH074 isolates replicated more efficiently in both MDCK and HEK293T cells, as compared with their respective avian-origin viruses, showing a significantly higher titer at each investigated time point (Fig. 1a). The control H9N2 V strain had an intermediate degree of replication, growing to a higher titer than the avian-origin viruses and a lower titer than the human-origin viruses. In avian DF1 cells, both human H10N8 JX346 and H7N9 GH074 viruses also grew to a higher titer than their respective avian-origin viruses and the control H9N2 virus at some time points (Fig. 1a). We intranasally infected 4 to 6-week-old female BALB/c mice with 10^6^ EID_50_ of each virus. Mice infected with JX346, GH074, or the lethal V virus showed symptoms of illness and weight loss starting from 2 days post-infection (d.p.i). Mice infected with avian-origin G1 or JX102 viruses did not display obvious clinical symptoms and weight loss, although mice infected with JX102 showed a slight weight loss (<5%) around 7 d.p.i. and then recovered (Fig. 1b, left). Two human-origin JX346 and GH074 viruses and the avian-origin V virus caused 100% case fatality rate in mice. However, all mice infected with JX346 died on 5 d.p.i., whereas animals infected the other 2 viruses showed a delayed fatality (after 7 or 8 d.p.i) (Fig. 1b, right). All 5 tested viruses replicated efficiently in the lungs of infected mice by 3 and 5 d.p.i. The human-origin H10N8, JX346, and H7N9 GH074 viruses grew to a significantly higher titer in mouse lungs when compared to their respective avian-origin JX102 and G1 viruses at both indicated time points (Fig. 1c, left). The control H9N2 V strain grew to a similar titer in mouse lungs as compared with the human-origin viruses. Our previous study showed that the H9N2 V virus is able to replicate in mouse brains^36^. We detected JX346 in the brains of infected mice at both early and late time points (2/3 mice in 3 d.p.i and 1/3 mice in 5 d.p.i.), by contrast to the H9N2 V virus. No virus was found in mouse brains infected with the avian-origin JX102 or both human- and avian-origin H7N9 viruses (Fig. 1c, right). Additionally, virus isolation was performed for tissue samples including kidney, liver and spleen collected from mice infected with the H7N9, H10N8 or H9N2 viruses. No virus was detected in these tissues (data not shown), indicating that the fatal H7N9, H10N8 and H9N2 viruses did not induce a systematic infection which is often observed with H5N1 influenza viruses^37^. Thus, both human-origin H10N8 JX346 and H7N9 GH074 viruses replicate more efficiently than their respective avian-origin viruses, and are lethal to mice without prior adaptation.

### PB2 contributes to the virulence of the human-original H10N8 virus in mice

**Figure 2:**
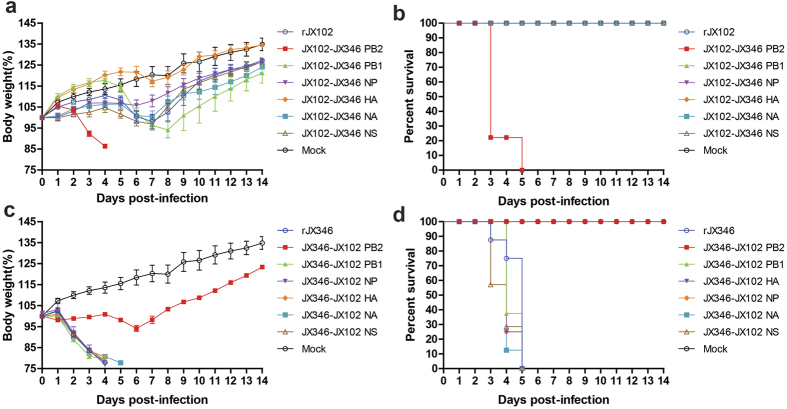
Pathogenicity of the H10N8 recombinant viruses in mice. Eight mice per group were intranasally inoculated with 10^6^ EID_50_ (in 50 μl) of the indicated viruses (or treated with PBS as a control). Body weight (**a,c**) and survival rate (**b,d**) was monitored daily for 14 days. Mice that lost more than 25% of their initial weight were euthanized. The percentage weight from each group and each time point are presented as means ± SD.

Both human-origin JX346 and avian-origin JX102 H10N8 viruses have identical PA and M genes and show higher homology (89.4% to 99.6%) in the other 6 gene segments^12^. To identify the genes that contribute to efficient replication and virulence of the human-origin JX346 H10N8 virus in mammalian hosts, a reverse genetic system for both JX346 and JX102 was developed and six recombinant viruses in the genetic background of the JX346 (or the JX102) with a single gene (PB2, PB1, NP, HA, NA or NS) from the JX102 (or the JX346) were generated and tested in mice. Eight mice of each group were intranasally infected with 10^6^ EID_50_ of the indicated viruses. The recombinant virus JX102-JX346PB2 containing the JX346-derived PB2 gene was virulent in mice, inducing severe weight loss (Fig. 2a) and 100% mortality, as compared with the rescued parental rJX102 and other 5 recombinant viruses (Fig. 2b). The JX102-JX346PB2 virus titer in mouse lungs was significantly higher than that of the parental rJX102 virus at 5 d.p.i (P < 0.05) (Table S1). The virus was only detected in mouse brains infected with this virus, not with the parental rJX102 or other 5 recombinant viruses (Table S1). Conversely, the recombinant virus JX346-JX102 PB2 containing the JX102-derived PB2 gene was attenuated in mice, inducing only a slight weight loss and no case fatality rate (Fig. 2c) when compared to the rescued parental rJX346 and other 5 recombinant viruses with a single gene from the JX102 virus (Fig. 2d). The JX346-JX102 PB2 virus titer in mouse lungs was significantly lower than that of the parental virus rJX346 and other 5 recombinant viruses with a single gene from the JX102 virus at both 3 and 5 d.p.i. (P < 0.01) (Table S1). No virus was detected in mouse brains infected with the recombinant virus JX346-JX102 PB2, whereas virus was found in the brains of mice infected with the parental virus rJX346 and other 5 recombinant viruses with a single gene from the JX102 virus at both 3 and 5 d.p.i. (Table S1). These results indicated that the PB2 gene is responsible for virulence and neural tropism of the human-origin H10N8 virus in mice.

### Lysine at position 627 of PB2 is not the only key virulence determinant of JX346 in mice

To identify amino acids in PB2 that contribute to the high virulence of JX346 in mice, we compared the PB2 sequences of JX346 and JX102 and found that there were eleven amino acid differences (Table 1). The amino acids at the eleven positions in both human-origin and avian-origin H7N9 and control H9N2 viruses were also compared (Table 1). PB2 627K, a well-characterized mammalian adaptation marker^22^,^25^, was present in all lethal strains tested in this study. To determine whether PB2 627K or other residues were responsible for the virulence of the human-origin H10N8 virus, two mutated virus with a single substitution in PB2 JX346-PB2 627E and JX102-PB2 627K were generated and tested in mice. As expected, the JX102-PB2 627K exhibited enhanced virulence, resulting in 100% case fatality rate and higher virus lung titers, as compared with the parental virus JX102 (Table 2). Furthermore, the virus was also detected in the brain of the infected mice (Table 2). However, unexpectedly, the mutated virus JX346-PB2 627E was still highly virulent, causing 100% case fatality rate and replicating efficiently in mouse lungs, similar to the parental virus JX346 although no virus could be detected in the brains (Table 2). To test whether 627E mutated to 627K in PB2, we sequenced the JX346-PB2 627E virus after replication in mice and found amino acid in position 627 of PB2 was still glutamate (Data not shown). Thus, lysine at position 627 (627K) is important but is not the only key determinant for virulence of the human-origin H10N8 virus.

### Other amino acids in PB2 responsible for the high virulence of JX346

**Figure 3:**
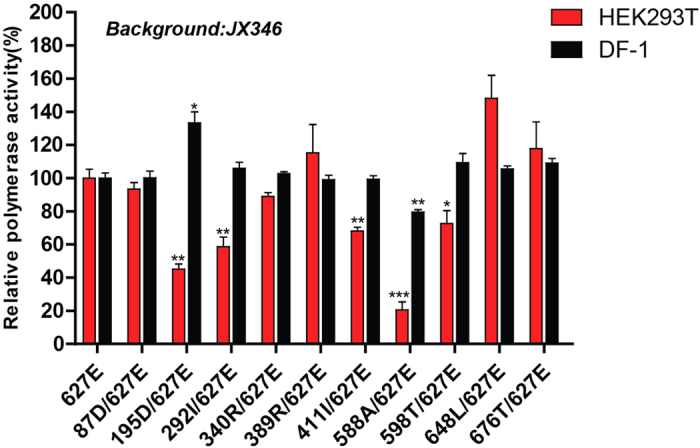
Polymerase activities of the JX346 PB2 with single or double substitutions. Human 293T or avian DF-1 cells were co-transfected with plasmids encoding the JX346 PB1, PA, NP, and PB2-627E or PB2 with double substitutions, together with a firefly luciferase reporter plasmid (driven by a human or an avian polymerase I promoter for use in human or avian cells, respectively), and a *Renilla* luciferase reporter plasmid (internal control). Cells were incubated for 24 h at 37 °C (HEK293T) or 39 °C (DF-1), and then firefly and *Renilla* luciferase activities were measured using a dual-luciferase assay. Polymerase activity was calculated by normalizing the firefly luciferase activity to the *Renilla* luciferase activity. The polymerase activity of the single 627E substitution in PB2 was set to 100% and the data are shown as mean relative polymerase activities ± SD (n = 3). (*p < 0.05, **P < 0.01, ***P < 0.001), according to a one-way ANOVA followed by a Dunnett’s test.

Since JX346-PB2 627E still exhibited a high pathogenicity in mice and in order to demine the influence of the other 10 amino acids besides 627K differing between JX346 and JX102 on polymerase activity, we performed a mini-genome assay using different genotypes of JX346 PB2 containing single substitutions (87E → D, 195G → D, 292V → I, 340K → R, 389K → R, 411V → I, 588V → A, 598V → T, 648V → L, 676M → T) combined with the K627E mutation in human 293T and avian DF-1 cells. The substituted residues 195D, 292I, 588A, and 598T combined with 627E in PB2 resulted in a significantly lower polymerase activity in HEK293T cells while no significant difference was observed for other substitutions when compared to the PB2 with a single mutant 627E (Fig. 3). Both 195D/627E and 292I/627E substitutions in PB2 decreased polymerase activity by ~50% and PB2 588A/627E reduced polymerase activity by ~80% (Fig. 3). In DF1 cells, the substitution 195D/627E in PB2 led to a significantly higher polymerase activity (140%), whereas the substitution 588A/627E induced a significantly lower polymerase activity (80%) when compared to the control PB2 with a single mutant 627E (Fig. 3).

Additionally, twelve mutant viruses that possessed the double substitutions in PB2 as described above in the genetic background of the JX346 virus were generated and tested in mice. In contrast to the parental JX346-wt and JX346-PB2 627E viruses, the mutant viruses including JX346-PB2 87D/627E, JX346-PB2 292I/627E, JX346-PB2 340R/627E, JX346-PB2 588A/627E, JX346-PB2 648L/627E, and JX346-PB2 676T/627E were less virulent (Table 2), whereas other mutant viruses including JX346-PB2 195D/627E, JX346-PB2 389R/627E, JX346-PB2 411I/627E, and JX346-PB2 598T/627E were still fully virulent. JX346-PB2 292I/627E and JX346-PB2 588A/627E did not cause any clinical signs (no weight loss) during infection and showed the lowest virulence, despite their efficient replication in mouse lungs (Table 2). The virus titers in lungs of mice infected with JX346-PB2 87D/627E, JX346-PB2 292I/627E, JX346-PB2 588A/627E, or JX346-PB2 648L/627E were also lower than those infected with the parental virus JX346 and JX346-PB2 627E viruses (Table 2). Taken together, these data indicate that 87E, 292V, 340K, 588V, 648V, and 676M in PB2 also play critical roles in the virulence of the H10N8 JX346 virus.

### Effects of 588A/V in PB2 on viral replication and pathogenicity of the H10N8 viruses

**Figure 4:**
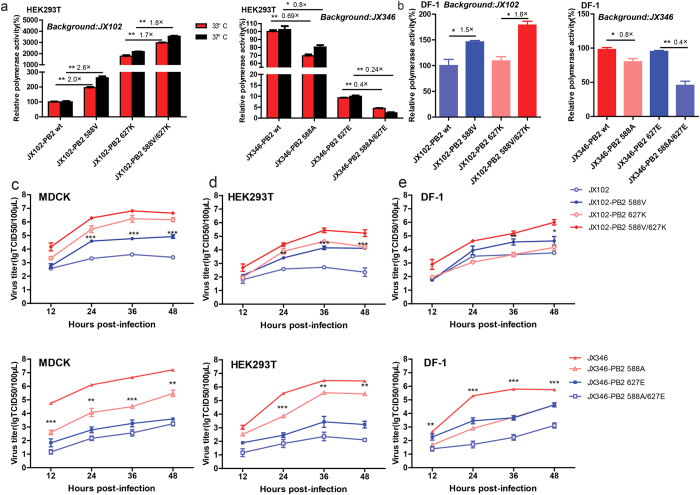
Polymerase activity and growth kinetics of JX102 or JX346 possessing different PB2 genotypes in human or avian cells. (**a**) HEK293T and (**b**) DF-1 cells were co-transfected with the RNP complexes composed of NP, PA, PB1 and wild type or mutant PB2 (single or double substitutions at position 588 and 627) of the JX102 or JX346 strain, together with a firefly luciferase reporter plasmid and *Renilla* luciferase reporter plasmid. Cells were incubated for 24 h at 37 °C, 33 °C (HEK293T) or 39 °C (DF-1). The polymerase activity of the wild type (JX346-wt and JX102-wt) was set to 100% and the data are presented as the mean luciferase activity from three independent experiments ± SD. Statistical significance was analyzed using an unpaired t test: *p < 0.05, **P < 0.01. **(c–e)** Growth kinetics of viruses: Confluent MDCK (**c**), HEK293T (**d**), and avian DF-1 (**e**) cells were infected with the indicated viruses at MOI of 0.01. Means of triplicate experiments are shown, with error bars representing the standard deviation. Statistical difference between the single PB2 588 mutant viruses compared with their respective parent viruses were labeled (*P < 0.05, **P < 0.01, ***P < 0.001)

While 87E, 292V, 340K, 588V, 627K, 648V, and 676M in PB2 play a critical role in the virulence the H10N8 JX346 virus, only the V588A substitution in PB2 resulted in a significantly lower polymerase activity in both human 293T and avian DF-1 cells (Fig. 3). Moreover, the results of mini-genome assays in HEK293T cells and DF1 cells showed that the polymerase activity was significantly enhanced due to introduction of PB2 A588V substitution into the JX102 PB2 when compared to the parental JX102-PB2 (Fig. 4a,b). Therefore, we investigated the effects of 588A/V in PB2 on H10N8 virus replication and virulence. We generated the single mutants JX346-PB2 588A, JX346-PB2 627E, JX102-PB2 588V, and JX102-PB2 627K and the double mutants JX346-PB2 588A/627E and JX102-PB2 588V/627K viruses in the genetic background of the human-origin JX436 and avian-origin JX102 viruses, respectively. In both MDCK and HEK293T cells, the substitution A588V markedly enhanced virus replication of JX102 and A588V/E627K slightly increased virus replication when compared to the E627K substitution (Fig. 4c,d). In DF1 cells, the mutant JX102-PB2 588V virus also replicated more efficiently than both wild type JX102 and mutant JX102-PB2 627K at later time points (Fig. 4e). By contrast, the PB2 substitution V588A markedly decreased virus replication of JX436 although it was not significant when compared to the substitution K627E and V588A/K627E in both MDCK and HEK293T cells (Fig. 4c,d). In DF-1 cells, three mutant viruses including JX346-PB2 588A, JX346-PB2 627E, and JX346-PB2 588A/627E showed decreased virus replication as compared with the parental JX346 virus, but the JX346-PB2 588A/627E grew to the lowest titer at later time points (Fig. 4e).

**Figure 5:**
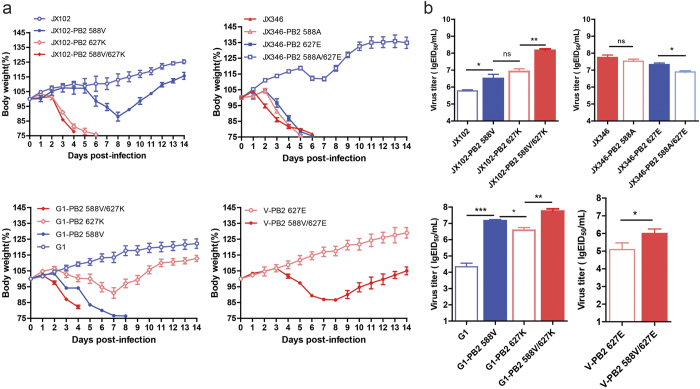
Infection and replication of H10N8, H7N9 and H9N2 viruses bearing different PB2 genotypes in mice. (**a**) Body weight changes post infection. Group of BALB/c mice (n = 11) were intranasally inoculated with 10^5^ EID_50_ (50 μl) of each virus as indicated. Body weight was monitored for 14 days and shown as means ± SD of 8 infected mice. (**b**) Virus replication in mouse lungs. Three mice from each group were euthanized at 3 days post infection and lung virus titers were determined. The values represent the log10 mean titers ± SD. Statistical significance was calculated using an unpaired t test: **P < 0.01; *P < 0.05; ns, no significance.

To investigate the effects of PB2 A588V on the virulence of the H10N8 viruses, we determined the 50% mouse lethal dose (MLD_50_) and virus replication in mice using mutant viruses. The single substitution A588V in PB2 enhanced the virulence of the avian-origin JX102 virus, evidenced by a reduced the dose of MLD_50_ and the efficient virus lung replication in infected mice when compared to the parental JX102 virus (Table 3 and Fig. 5), although it did not result in a markedly increased virulence when compared to introduction of substitution E627K or A588V/E627K into the PB2. By contrast, the single substitution V588A, similar to K627E in PB2 for the human-origin JX346, had a slight effect on the MLD_50_ value, virulence, and virus replication in lungs of infected mice (Table 3), but the substitution V588A coupled with K627E in PB2 attenuated the parental JX346 virus, evidenced by a significantly enhanced MLD_50_ value and no case fatality rate in infected mice (Table 3 and Fig. 5). Moreover, histopathological analysis of the lung tissues showed that mice infected with JX102-PB2 588V, JX102-PB2 627K or JX102-PB2 588V/627K had serious pathological inflammatory injury when compared to those from mice infected with the JX102 virus (Fig. S1). These data revealed that PB2 588V alone, or combined with 627K contributed to virulence and virus replication and adaptation of H10N8 avian influenza virus in mice.

### Mutation of A588V in PB2 enhances polymerase activity, virus replication, and virulence of avian-origin H7N9 and H9N2 viruses

**Figure 6:**
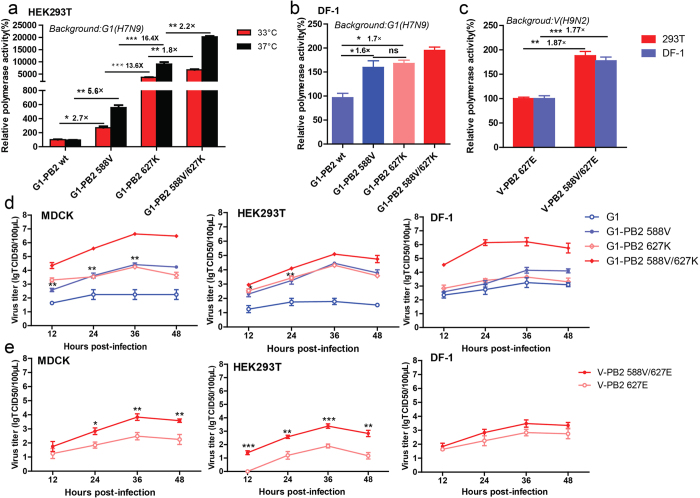
Polymerase activity and growth kinetics of avian-origin H7N9 (or H9N2) viruses possessing different PB2 genotypes. (**a–c**) Polymerase activity. HEK293T and DF1 cells were co-transfected with the RNP complex of the H7N9 virus (**a**,**b**) or H9N2 virus (**c**) with different PB2 genotypes. The data are the mean of three independent experiments ± SD. **(d,e)** Growth kinetics of the avian-origin H7N9 G1 virus or H9N2 virus and their variants possessing PB2 588V, 627K, or 588V/627K were compared in MDCK, HEK293T and avian DF-1 cells. Means of triplicate experiments are shown, with error bars representing the standard deviation. Statistical difference between the single PB2 588 mutant viruses compared with their respective parent viruses were labeled (*P < 0.05, **P < 0.01, ***P < 0.001).

To investigate whether the effects of mutation A588V on virulence and virus replication also apply for the emerging zoonotic H7N9 virus, the mutant viruses with single (A588V or E627K) or double (A588V/E627K) substitutions in PB2 were generated in the genetic background of the H7N9 avian-origin G1 virus. The results of mini-genome assays in HEK293T cells showed that the polymerase activity was significantly enhanced due to the introduction of PB2 A588V substitution into the G1 PB2 at both 33 °C and 37 °C, as compared with the parental G1-PB2, although it did not result in a similar level of polymerase activity induced by the substitution of E627K or A588V/E627K (Fig. 6a). In DF1 cells, both G1-PB2 A588V and G1-PB2 E627K substitutions exhibited a same level of polymerase activity that was significantly higher than that of the parental G1-PB2 (Fig. 6b). Consistent with the results of polymerase activity, both G1-PB2 588V and G1-PB2 627K mutant viruses replicated more efficiently than the wild type parental G1 virus in both MDCK and HEK293T cells, but did not grow to the same high titers as the double mutant G1-PB2 A588V/E627K virus (Fig. 6d). Both G1-PB2 588V and G1-PB2 627K mutant viruses replicated to a comparable level in DF1 cells as the wild type parental G1, but the double mutant G1-PB2 588V/627K virus still replicated to significantly higher titers at each time point as compared with the other 3 viruses (Fig. 6d).

Both the single G1-PB2 588V and double mutant G1-PB2 588V/627K substitutions in these H7N9 mutant viruses resulted in enhanced virulence in mice, evidenced by the reduced MLD_50_ value (Table 3), weight loss and case fatality rate when compared to the parental G1 virus (Fig. 5). To our surprise, the substitution G1-PB2 627K did not enhance virulence, as has been demonstrated for other avian influenza viruses^36^, although it still caused weight loss (Fig. 5a). Virus replication in the lungs of infected mice were consistent with those observed in tested mammalian cells, i.e., mutant viruses showed higher titers than the parental G1 virus (Fig. 5b).

We have shown that the H9N2 V-PB2 627E is not virulent in mice^36^. We generated a mutant V-PB2 A588V/K627E virus in the genetic background of the V-PB2 627E virus to investigate the effects of the PB2 A588V substitution on virulence. The substitution A588V in PB2 significantly increased the polymerase activity in both HEK293T and DF1 cells (Fig. 6c) and the mutant V-PB2 588V/627E replicated more efficiently than V-PB2 627E in both MDCK and HEK293T cells, although they exhibited no significant difference in DF1 cells (Fig. 6e). The mutant V-PB2 588V/627E virus was able to cause disease (obvious weight loss) and replicate more efficiently in the lungs of mice than the control V-PB2 627E virus (Fig. 5a), although it did not cause case fatality rate (MLD_50_ > 10^6^EID_50_) (Table 3). Taken together, the substitution A588V in PB2 also enhanced the polymerase activity in both human and avian cells and the virulence of avian-origin H7N9 and H9N2 viruses.

### Proportion of H9N2, H7N9, and H10N8 viral isolates possessing residue 588V in PB2

To determine the frequency of residue 588V in PB2 among human or avian H7N9, H10N8, and H9N2 influenza viruses, the available full-length PB2 sequences of these subtypes obtained from NCBI Influenza Virus Resource database (http://www.ncbi.nlm.nih.gov/genomes/FLU/FLU.html) and GISAID (http://platform. gisaid.org) were analyzed. There were four variable amino acids (A, V, T, I) at this position in PB2 and the Alanine (A) was the most common amino acids (Table 4), considered as a conserved residue among avian influenza viruses^38^. The avian H9N2 isolates possessing a residue 588V occupied just a small fraction (4.3%) prior to 2012, but increased to 21.9% since 2013 in which the first human case infected with the H7N9 virus occurred. Human-origin H7N9 viruses encoding PB2 588V were increased from 0% in 2013 to 24.2% (8/33) in 2014 (Table 4) despite few (1.9%) avian-origin H7N9 virus encoding PB2 588V. The PB2 of one human H9N2 isolate A/Lengshuitan/11197/2013 also encodes 588V (while encoding 627E in PB2)^39^. In addition, there were 38.1% avian-origin H10N8 viruses that encode 588V in PB2 and 100% of human-origin H10N8 viruses encoding 588V in PB2. Among the human isolates bearing 588V in PB2, we found that 75% (6/8) of H7N9 and 66.6% (2/3) of H10N8 viruses contain both PB2 588V and 627K. These data indicate that H7N9, H10N8, and H9N2 subtype viruses encoding 588V in PB2 have increased in abundance since the first identification of human infections in 2013.

## Discussion

The recently emerged H7N9 and H10N8 viruses are able to induce severe respiratory illness and high case fatality rate (35%) in humans, but they do not cause obvious disease and mortality in chickens^40^. The severe infection was consistent with our mouse experiments of both human-origin H7N9 and H10N8 viruses that showed high virulence and efficient virus replication in mice. Fortunately, these viruses have not acquired the ability of sustained human-to-human transmission, although limited human-to-human transmission cannot be excluded in a few clusters of H7N9 infections (http://www.who.int/influenza/en/). No further human H10N8 infections cases have been reported, most likely because the H10N8 virus has not adapted to mammalian hosts and still prefers to bind avian-like receptors^19^,^20^,^41^,^42^.

To date, it remains unclear why and how avian H7N9 and H10N8 viruses acquire the ability to cross the species barrier to infect humans. A recent study has shown that the internal genes PB2, M, and NP from the endemic H9N2 virus^11^,^12^,^43^ contribute to the high infectivity of the H7N9 virus in humans^32^. Our studies here also indicate that PB2 is the key virulence determinant for the human H10N8 JX346 and H7N9 G1 viruses. It should be noted that the PB2 of JX346 originates from local endemic H9N2 viruses circulating in chickens, whereas the PB2 of the chicken-origin H10N8 JX102 virus is derived from the H7 virus in ducks^12^. Replacement with the JX102 PB2 resulted in avirulence of JX346, highlighting that PB2 of the currently circulating H9N2 viruses is important for the emergence of novel zoonotic influenza viruses such as H7N9 and H10N8. Furthermore, recent studies revealed that circulating H7N9 viruses still continue to reassort with endemic H9N2 and other subtype influenza viruses, leading to different genetic linages^44^,^45^. Therefore, the mammalian adapted PB2 from the H10N8 or H9N2 viruses could be transmitted to other subtype viruses such as H5N1 and H6N6 through reassortment, resulting in zoonotic viruses that might cause epidemics and potential pandemics in humans.

Many adaptive mutations in PB2, such as E627K^46^, D701N^29^, K526R^30^, and T271A^38^ have been proven to be important for different avian influenza viruses to break the host species barrier to infect mammals^21^. These substitutions were also confirmed to be important for novel H7N9 influenza virus to adapt mammalian hosts^34^,^35^. In our study, besides 627K, six residues 87E, 292V, 340K, 588V, 648V, and 676M in PB2 contributed to the high virulence of the human H10N8 JX346 virus in mice. Interestingly, among these residues only V292I and V588A substitutions resulted in a significantly lower polymerase activity in human cells (Fig. 3); however, whether or not their virulence correlates with the polymerase activity remains unclear and needs to be investigated in future research. Importantly, the residues 292V, 648V, and 676M in PB2 exist in currently circulating H9N2 and H7N9 viruses^47^. Noticeably, the amino acid 292V/I has been shown to be conserved in human and avian isolates^48^, therefore, 292V might be another important residue for mammalian adaptation of avian influenza viruses. Moreover, some amino acid changes in PA have been demonstrated to affect the replication and virulence of H7N9 virus in mammals^49^. Our studies also demonstrate that some amino acid changes in PB2 are important for the virulence of avian influenza viruses including H10N8 virus in mammalian host. These data suggest that the evolutionary changes in PB2 of H9N2 or H7N9 viruses may facilitate virus mammalian host adaptation and enhance virus replication and transmission in mammals.

Neural tropism of the influenza viruses (Fig. 1C) is an interesting topic. Our results showed that virus was only detected from brains of mice infected with the viruses with the PB2 627K and not from those infected with the 627E mutant viruses (Table 2 and S1). This is consistent with observations reported in a previous study using the H5N1 virus^50^. Furthermore, no virus was detected from brains of mice infected with H10N8 JX346 mutant viruses which have 627E/588V in the PB2. Collectively, this data indicates that influenza virus neural tropism may be related to the 627K in PB2, not the 588V in the PB2. However, the mechanisms regarding neural tropism remain unclear and needs to be investigated.

The conserved structure of the newly defined 627-domain in PB2^51^,^52^,^53^,^54^ has been shown to be essential for the influenza polymerase function, replication, and host adaptation. Residues 590 and 591 located in this domain are a key adaptive strategy of the 2009 pandemic H1N1 influenza virus in humans^55^. Here we showed that residue 588V, located in the PB2 627-domain near the polymorphic 590 and 591 residues, is important for H7N9 and H10N8 virus replication and virulence. Additionally, previous studies had reported that the mutation T588I or A588T in PB2 could enhance the virulence of 2009 pandemic H1N1 influenza virus^56^ or H5N1 avian influenza virus^31^. In our study, the mutation A588V in PB2 enhanced the virulence of avian H7N9, H10N8, and H9N2 influenza viruses. Together, these data indicate the importance of the 588 position in PB2 for polymerase activity, replication, and virulence of different subtypes of avian influenza viruses in mammals, suggesting that it is another mammalian adaption marker for avian influenza viruses. However, when introduced the PB2 588A into JX346 virus (encoding PB2 627K) has a minor effect in the virulence (Table 3), it may indicate that the virulence factors of influenza virus are complex and the high virulence may be the outcome of the action of multiple amino acids in the viral proteins. It is interesting that the PB2-588V also enhanced the viral replication and polymerase activity in DF1 cells (Figs 4 and 6); however, *in vitro* results may not represent virus biological features such as virulence *in vivo*.

Although many adaptive mutations in PB2 have been identified, the underlying mechanism of influenza host adaptation is unclear. Differing side chains in residues at 588 may affect PB2 structure and its interaction with other viral or host proteins, which is important for influenza virus to adapt to new hosts^57^. Previous studies have shown that D701N and E627K may influence the interaction of PB2 with different isoforms of importin-α to adapt mammalian hosts^58^,^59^ and a recent study showed that the PB2-627K resists the signaling-independent antiviral effect of RIG-I by increasing nucleocapsid stability^60^. How the residue 588V affects virus replication and virulence needs to be investigated in the future.

It is interesting that the H7N9 G1 mutant virus, G1-PB2 627K, displayed relatively low virulence in mice, even lower than the 588 mutant virus, G1-PB2 588V (Fig. 5a), although the PB2 627K has been demonstrated to be an important virulence maker for numerous influenza viruses. This phenomenon was also observed in other human H7N9 isolates such as A/Anhui/1/2013 and A/Shanghai/2/2013 that have PB2 588A/627K reported in a previous study^61^. It should also be noticed that the H7N9 A/Shanghai/1/2013 virus bearing PB2 588A/627K in the previous study showed high virulence, similar to the H7N9 GH074 virus used in our study (Fig. 1). These data indicate that *i)* the effect of the PB2 627K on influenza viruses may be strain-dependent, similar to data previously reported with H5N1^62^; *ii)* the PB2 627K mutation is not the only key factor for mammalian adaption of the novel H7N9 influenza virus and other adaptive strategies to mammalian hosts may be employed for this virus (many adaption mutations already exist in this virus^47^), such as the A588V identified in our study. The G1-PB2 588V showed more enhanced virulence than the G1-PB2 627K in mice although both viruses displayed similar growth dynamics in tested cell lines, indicating that enhanced virulence might not always correlate with better virus replication and increased polymerase activity in cell cultures. Host immune responses or other pathological factors are also important for the virulence besides virus replication capacity as reported in the previous study^24^.

Genetic analysis showed that the proportion of influenza isolates encoding 588V in PB2 among avian or human H7N9, H10N8, and H9N2 viruses are increasing. Notably, many human H7N9 (75%) and H10N8 (66.6%) isolates contain the combination of PB2 588V/627K which has been demonstrated to have substantial effects on virus replication capacity and virulence in both H10N8 and H7N9 viruses (Figs 4,5 and 6), indicating that the PB2 588V is essential for mammalian adaptation.

In summary, PB2-588V identified in our study is important for polymerase activity, virus replication, and virulence in mice, indicating that it may be a new marker of mammalian adaption for the avian influenza virus. Therefore, attention should be given to this new marker during surveillance of avian influenza viruses.

## Materials and Methods

### Viruses and cells

MDCK, Human embryonic kidney 293T (HEK 293T) and avian cells DF-1 were grown in Dulbecco’s modified essential medium (DMEM) with 10% fetal bovine serum and antibiotics. The H10N8 A/Jiangxi-Donghu/346-1/2013 (JX346) virus was isolated from a patient with a fatal infection and A/chicken/Jiangxi/102/2013 (H10N8) (JX102) was isolated from healthy chickens in a live poultry market (LPM) that the patient had visited. The phylogenetic and genetic properties of both JX346 and JX102 have been described^12^. The H7N9 A/Guangdong/GH074/2013 (GH074) was isolated from a patient in Guangdong and A/chicken/Guangdong /G1/2013 (H7N9) (G1) was isolated from healthy chickens in a LPM in Guangdong. The H9N2 A/chicken/Guangdong/V/2008 (V) was isolated from a chicken in a poultry farm in Guangdong. Viruses were propagated in 9- to 11-day-old specific pathogen free (SPF) embryonated chicken eggs and stored at −70 °C until they were used.

### Plasmid construction and reverse genetics

Eight gene segments from the JX346, JX102, and G1 strains were cloned into a pHW2000 plasmid system and were confirmed using DNA sequencing. Reverse genetic systems for V and V-627E were described previously. The PB2 mutant constructs were produced by site-directed mutagenesis PCR and sequenced. The recombinant and mutant viruses were generated using reverse genetics. HEK293T cells monolayers in 6-well plates were transfected at 80–90% confluency with 4 μg of the eight plasmids (500 ng of each plasmid) by using Lipofectamine 3000 (Invitrogen) according to the manufacturer’s instructions. DNA and transfection reagent were mixed (8 μl p3000 reagent and 7.5 μl Lipofectamine 3000 per well), incubated at room temperature for 5 min, and added to the cells. Six hours later, the mixture was replaced with Opti-MEM (GIBCO) containing 0.2% bovine serum albumin and 1 μg/ml TPCK trypsin. After 48 h, the supernatant was harvested and injected into SPF embryonated eggs for virus propagation. Viruses were titrated in embryonated eggs using hemagglutination assays and sequenced.

### Mouse experiments

Groups of fourteen 4- to 6-week-old female BALB/c mice, obtained from the Vitalriver Company in Beijing, were anesthetized with CO_2_ and inoculated intranasally with indicated viruses. Body weight and clinical symptoms were monitored daily for 14 days after infection. The mice were euthanized if they lost more than 25% of their initial body weight. To test virus replication in organs, three mice in each group were euthanized on days 3 and 5 p.i., respectively. Tissue samples including brain, lung, kidney, liver and spleen, from each euthanized mouse were collected for virus titration by an EID_50_ assay. To determine MLD_50_ of each virus, three mice per group were anaesthetized with CO_2_ and inoculated intranasally with 10-fold serially diluted each virus in a 50 μl volume (the dose ranges from 10^1^ to 10^6^ EID_50_) The MLD_50_ of each virus was calculated using the method of Reed and Muench.

### Minigenome reporter assays

For polymerase activity assays, RNP complexes composed of PA, PB1, PB2 and NP (200 ng each) were mixed with a luciferase reporter plasmid (200 ng) and a thymidine kinase promoter-Renilla luciferase reporter plasmid (pRL_TK) construct (20 ng), then co-transfected into HEK293T cells in a 12-well plate with Lipofectamine3000 (Invitrogen, Carlsbad, CA), and incubated at 33 °C or 37 °C for 24 h. Luciferase production was assayed using the dual-luciferase reporter assay system (Promega) according to the manufacturer’s instructions. Polymerase activity was normalized to Renilla expression. For the luciferase reporter assays in DF-1 cells, a luciferase reporter containing a chicken RNA polymerase I (Pol I) promoter was constructed as described previously^63^, and the transfected DF-1 cells were incubated at 39 °C for 24 h. All results shown are the means with standard deviations from three independent experiments.

### Virus growth kinetics

Confluent MDCK, HEK293T or DF-1 cells were infected at a multiplicity of infection (MOI) of 0.01 TCID_50_/cell for 1 h at 37 °C. After 1 h incubation, the cells were washed twice, and then incubated with DMEM containing 0.2% bovine serum albumin (BSA) and TPCK trypsin (1.0 μg/ml) at 37 °C with 5% CO_2_. Culture supernatants were collected at indicated time points and viral titers were determined by the 50% tissue culture infective dose (TCID_50_) assay in MDCK cells.

### Ethics statement and Biosafety

All animal experiments involving recombinant H7N9 and H10N8 viruses were reviewed and approved by the Institutional Animal Care and Use Committee at South China Agricultural University (SCAU) and were carried out in accordance with the approved guidelines. All experiments were conducted in biosafety level 3 laboratories and animal facilities at SCAU.

### Statistical analyses

Differences between experimental groups were determined by using an unpaired t-test and analysis of variance (ANOVA) in the GraphPad Prism software (GraphPad Software Inc.).

## Additional Information

**How to cite this article**: Xiao, C. *et al*. PB2-588V promotes the mammalian adaptation of H10N8, H7N9 and H9N2 avian influenza viruses. *Sci. Rep.* **6**, 19474; doi: 10.1038/srep19474 (2016).

## Supplementary Material

Supplementary Information

## Figures and Tables

**Table 1:** Amino acid differences between JX346 and JX102 PB2 proteins and comparison with H7N9 and H9N2 PB2.

**Virus**	**Amino acid position**										
	**87**	**195**	**292**	**340**	**389**	**411**	**588**	**598**	**627**	**648**	**676**
A/Jiangxi-Donghu/346-1/2013(H10N8)	**E**#	**G**	V	K	K	**V**	**V**	V	K	V	M
A/chicken/Jiangxi/102/ 2013 (H10N8)	D	D	I	R	R	I	A	T	E	L	T
A/Guangdong/GH074/2013(H7N9)	D	D	V	R	K	I	A	V	K	V	M
A/chicken/Guangdong/G1/2013(H7N9)	D	D	V	R	K	I	A	V	E	V	M
A/chicken/Guangdong/V/2008(H9N2)	D	D	V	K	R	I	A	T	K	L	T

^#^Boldface letters indicate the JX346-PB2 residues that differ from the others.

**Table 2:** Virulence and virus replication of the mutant H10N8 viruses with PB2 627E/K in mice.

**Virus**	Infectiondose (EID_50_)	Survival(%)#	Maximumweight loss#	Virus titer in organs*(Log_10_ EID50/ml)	
				**Lung**	**Brain**$
JX102-wt	10^5^	100	0%	6.0 ± 0.4	0/3
JX102-PB2 627K	10^5^	0	23%	7.0 ± 0.4^b^	1/3(1.25)
JX346-wt	10^5^	0	24%	7.4 ± 0.1	2/3(2.5, 3)
JX346-PB2 627E	10^5^	0	22%	7.2 ± 0.1^d^	0/3
JX346-PB2 87D/627E	10^5^	100	7%	6.7 ± 0.1^b^	0/3
JX346-PB2 195D/627E	10^5^	0	25%	6.8 ± 0.2	0/3
JX346-PB2 292I/627E	10^5^	100	0%	6.3 ± 0.2^b^	0/3
JX346-PB2 340R/627E	10^5^	100	5%	6.8 ± 0.3	0/3
JX346-PB2 389R/627E	10^5^	0	24%	7.3 ± 0.1	0/3
JX346-PB2 411I/627E	10^5^	0	25%	7.2 ± 0.3	0/3
JX346-PB2 588A/627E	10^5^	100	0%	6.4 ± 0.1^b^	0/3
JX346-PB2 598T/627E	10^5^	0	24%	7.0 ± 0.5	0/3
JX346-PB2 648L/627E	10^5^	100	5%	6.2 ± 0.3^c^	0/3
JX346-PB2 676T/627E	10^5^	87.5	15%	6.9 ± 0.2	0/3

^*^Groups of mice were infected with 10^5^ EID50 of each virus. Three mice from each group were euthanized, the brains and lungs were homogenized in 1 ml of phosphate-buffered saline for virus titration in eggs at 3 days p.i.

^#^Body weight and survival of infected mice were monitored for 14 days. The survival and maximum weight loss were calculated.

^§^The number of mice detected with viral infection in brain/total (titer[s] in individual positive mouse).

^b^Significantly different (P < 0.01) from titers in lungs of mice that received the corresponding parental virus.

^c^Significantly different (P < 0.05) from titers in lungs of mice that received the corresponding parental virus JX346-PB2 627E.

^d^Not significantly different (P > 0.05) from titers in lungs of mice that received the corresponding parental virus JX346-PB2 627E.

**Table 3:** MLD_50_ of H10N8, H7N9, and H9N2 PB2 mutant viruses in BALB/c mice.

**Subtype**	**Virus name**	**MLD50 (log**_**10**_**EID**_**50**_)
H10N8	JX102-wt	>6
	JX102-PB2 588V	5.5
	JX102-PB2 627K	2.75
	JX102-PB2 588V/627K	2.25
	JX346-wt	2.25
	JX346-PB2 588A	2.5
	JX346-PB2 627E	3.75
	JX346-PB2 588A/627E	5.5
H7N9	G1-wt	>6
	G1-PB2 588V	4.5
	G1-PB2 627K	>6
	G1-PB2 588V/627K	2.75
H9N2	V-PB2 627E	>6
	V-PB2 588V/627E	>6

**Table 4:** Amino acids at position 588 in PB2 of human and avian H9N2, H7N9, and H10N8 viruses in different years.

	**H9N2**			**H7N9**			**H10N8**	
	**Avian**		**Human**	**Avian**	**Human**		**Avian**	**Human**
Residue	−2012(n = 530)	2013-(n = 96)	−2014(n = 8)	2013-(n = 54)	2013$(n = 85)	2014-(n = 33)	2013$-(n = 21)	2013-(n = 3)
A	429 (81%)#	74(77.1%)	7(87.5%)	53(98.1%)	85(100%)	25(75.8%)	13(61.9%)	0
V	23 (4.3%)	21(21.9%)	1(12.5%)	1(1.9%)	0	8(24.2%)	8(38.1%)	3(100%)
T	36 (6.8%)	1(1%)	0	0	0	0	0	0
I	42 (7.9%)	0	0	0	0	0	0	0

^#^The number of isolates possessing indicated amino acid and the percentage of total available isolates.

^$^Dates for collecting PB2 sequences of both H7N9 and H10N8 viruses start when the first human case infected with H7N9 was identified.
