# Supplementary material for: PB2-588 V promotes the mammalian adaptation of H10N8, H7N9 and H9N2 avian influenza viruses
Source: Sci Rep. 2016 Jan 19;6:19474. doi: 10.1038/srep19474 (PMC4726052; doi:10.1038/srep19474)
Supplement: Supplementary Information [file srep19474-s1.pdf]

PB2-588V promotes the mammalian adaptation of H10N8, H7N9 and H9N2 avian influenza viruses

Chencheng Xiao[1*], Wenjun Ma[2*], Na Sun[1*], Lihong Huang[1], Yaling Li[1], Zhaoyong Zeng[1], Yijun Wen[1], Zaoyue Zhang[1] , Huanan Li[1],Qian Li[1], Yuandi Yu[1], Yi Zheng[1], Shukai Liu[1], Pingsheng Hu[1], Xu Zhang[1], Zhangyong Ning[1], Wenbao Qi[1†],and Ming Liao[1†]

1. National and Local Joint Engineering Laboratory for Medicament of Zoonosis Prevention and Control, College of Veterinary Medicine, South China Agricultural University, Guangzhou, Guangdong Province 510642, People's Republic of China.

2. Department of Diagnostic Medicine/Pathobiology, Kansas State University, Manhattan, KS 66502, USA

[*]Chencheng Xiao, Wenjun Ma and Na Sun contributed equally to this paper.

[†]Correspondence: Wenbao Qi, South China Agricultural University, No. 483 Wushan Road, Tianhe District, Guangzhou, China 510642; Email: qiwenbao@scau.edu.cn

Ming Liao, South China Agricultural University, No. 483 Wushan Road, Tianhe District, Guangzhou, China 510642; Email: mliao@scau.edu.cn

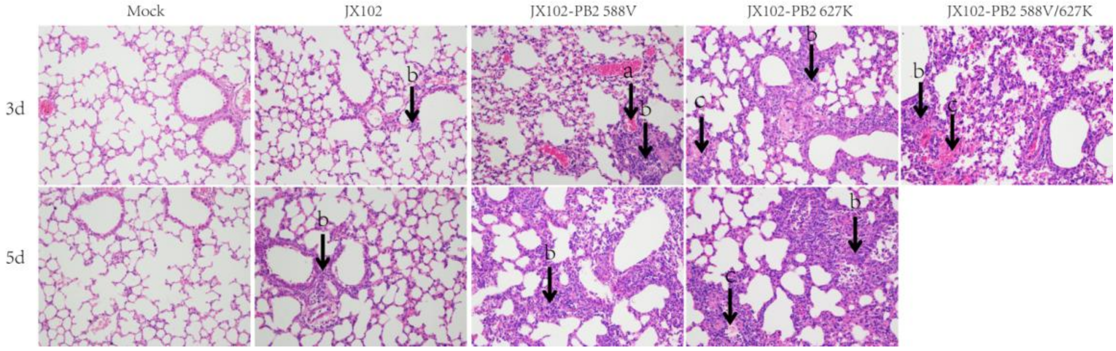

Fig S1 Histopathological change of the lung from mice infected with the JX102 and its PB2 mutant viruses on days 3 and 5 p.i. Lungs of infected mice were fixed with formalin, embedded in paraffin, and stained with hematoxylin and eosin. The H&E staining result for the mice infected with JX102-PB2 588V/627K is not possible since all animals died prior to 5 days post infection. The images were obtained at a magnification of 200.Arrow a: alveolar wall capillary hyperemia; arrow b: infiltration of inflammatory cells; arrow c: hemorrhage.

Table S1. Virus replication of the recombinant H10N8 viruses in mouse lungs and brains.

Virus	Virus titer in organs ($LogEID_{50}$/ml)			
	Lung*		Brain	
	3dpi	5dpi	3dpi	5dpi
rJX346	7.5±0.3	7.4±0.2[b]	2/3(3.33, 1.5)[§]	2/3(1.5,1.95)
JX346-JX102 PB2	5.6±0.2[b]	6.5±0.3[b]	0/3	0/3
JX346-JX102 PB1	7.7±0.3	7.3±0.2	2/3(1.25, 1.5)	1/3(1.5)
JX346-JX102 NP	7.8±0.4	7.1±0.3	3/3(2.2±1.2)[#]	2/3(3.0, 2.5)
JX346-JX102 HA	7.6±0.2	7.1±0.3	2/3(1.25, 1.5)	0/3
JX346-JX102 NA	7.3±0.3	7.4±0.2	2/3(1.5, 1.5)	1/3(2.5)
JX346-JX102 NS	7.7±0.4	7.8±0.2	1/3(2.25)	1/3(2.75)
rJX102	6.5±0.3	6.6±0.2[c]	0/3	0/3
JX102-JX346 PB2	7.0±0.3	7.3±0.2[c]	2/3(2.5, 2.75)	1/3(1.95)
JX102-JX346 PB1	6.5±0.4	7.0±0.25	0/3	0/3
JX102-JX346 NP	6.8±0.2	6.5±0.2	0/3	0/3
JX102-JX346 HA	6.8±0.3	6.9±0.2	0/3	0/3
JX102-JX346 NA	6.4±0.2	6.4±0.4	0/3	0/3
JX102-JX346 NS	6.6±0.4	7.0±0.4	0/3	0/3

* All lungs of the mice in each group had detectable virus (3/3).

§The number of mice that was detected virus with infection in brain/total (titer[s] in individual positive mice)

b Significantly different (P<0.01) from titers in lungs of mice that received the corresponding parental virus.

C Significantly different (P<0.05) from titers in lungs of mice that received the corresponding parental virus.

when all three animals in a treatment group had positive virus titration results for the brain, the mean±standard deviation is reported in parentheses (rather than results for individual animals).
